# Stokes-Adams Disease

**Published:** 1905-05-06

**Authors:** 


### STOKES-ADAMS DISEASE.

In 1827 Adams published a case of apoplectiform seizures in association with a peculiar slowness of the pulse. Autopsy showed that the patient suffered from fatty degeneration of the myocardium. This combination was subsequently studied by Stokes, who, in 1846 published observations confirmatory of its dependence upon myocardial degeneration. Halberton two years later recorded a case where this syndrome followed an injury to the cervical spinal cord, and later observers have demonstrated the connection of these symptoms with lesions compromising the integrity of the vagus nucleus in the bulb. For the unhappy name we are indebted to Huchard, who christened as the "Stokes-Adams syndrome" a combination of bradycardia with vertiginous attacks or seizures, either epileptic or apoplectiform.

This historical summary is drawn from a paper by Dr. A. Luco,[1] in which he details a case of the kind. The patient was a single woman of 55, who, nine years before she came under observation, began to suffer from pains in the extremities, most severe at night. These gradually disappeared in the course of six months, and she remained well until an attack of influenza four years later left her subject to attacks of palpitation and dyspnœa. During the following year she suffered much from headache (again most severe at night) and vomiting seizures of a cerebral type. Shortly before the illness which brought her under observation, she developed œdema, which began about the ankles and presently became generalised. This disappeared under treatment, but she commenced to experience disturbances of equilibrium. In these vertiginous attacks she occasionally fell, sometimes merely unconscious for a few minutes, sometimes exhibiting generalised convulsive movements. Her sleep became disturbed, and broken by frequent nightmares. She would wake nightly at the same hour, with a sense of great oppression, accompanied by tremor and chill, and followed by præcordial pain, the whole attack lasting for about 20 minutes. One day she fell to the ground unconscious, and remained so until the following day. She was then found to have complete paralysis of the left upper extremity, and of the left side of the face. There was considerable interference with her speech, and though nothing abnormal was noted about the lower extremities at first, it was clear, later, that the movements of the left leg were distinctly less forcible than those of the other side. The urine was free from albumin and sugar, and the optic discs somewhat swollen. The condition of the heart was remarkable. The heart-sounds were feeble, though otherwise normal in quality, but the pulse-rate was extremely slow. The heart-beats were quite regular, but amounted on the first day to only 35 per minute. A few days later movement began to return to the paralysed limb, but at the date of writing it remained weak; the headache and vomiting abated to a large extent, but the patient had completely lost her memory and showed some signs of mental confusion. The author quotes Brissaud to the effect that this syndrome merely serves to localise the lesion, for the symptoms are the same whether they be due to a gumma, or a tuberculous mass, or chronic meningitis, or atheroma of the bulbar vessels.

1 Revista Méd. de Chile, Sept. 1904.

